# Editorial: Neurobiology of spontaneous object exploration in recognition memory

**DOI:** 10.3389/fnbeh.2023.1184935

**Published:** 2023-05-03

**Authors:** Owen Chao, Flávio F. Barbosa, Marion Inostroza, James A. Ainge, Jay-Shake Li

**Affiliations:** ^1^Department of Biomedical Sciences, Medical School, University of Minnesota, Duluth, MN, United States; ^2^Department of Psychology, Federal University of Paraíba, João Pessoa, Paraíba, Brazil; ^3^Institute of Medical Psychology and Behavioral Neurobiology, University of Tuebingen, Tuebingen, Germany; ^4^School of Psychology and Neuroscience, University of St Andrews, St Andrews, United Kingdom; ^5^Department of Psychology, National Chung Cheng University, Minxiong, Taiwan

**Keywords:** object recognition, episodic memory, protein kinase Mζ (PKMζ), dopamine, BDNF

Spontaneous object exploration is an innate behavior that many species exhibit, which refers to the investigation of objects within the environment without explicit goals or rewards. Animals engage naturally in exploratory behaviors toward objects with novel features, such as new identity, location, and contextual associations, as compared to objects with familiar ones. This suggests that the propensity to objects with novel features is influenced by cognitive processes of perception, attention, and learning and memory, as the novelty preference is driven by remembrance of the familiar objects. Researchers have utilized spontaneous object exploration, which involves no extensive training, incentives or deterrents, and one-trial learning and retrieval, to develop paradigms for studying recognition memory of objects, places, contexts, their associations, and even episodic-like memory, mimicking human daily experiences. While significant evidence has been accumulated over the past two decades regarding the neural mechanisms of recognition memory, questions remain: How animals develop the ability to remember and distinguish between different mnemonic features of objects? Does sex play a role in recognition memory? How do distinct neurochemical systems influence recognition memory? Essentially, what are the cellular and molecular mechanisms involved in memory processing within the hippocampus (HPC) and perirhinal cortex (PRC), the two key regions for recognition memory?

We are delighted to present the Research Topic “*Neurobiology of spontaneous object exploration in recognition memory*,” published in the journal *Frontiers in Behavioral Neuroscience*. This Research Topic covers recent advancements in the neurochemical, cellular, and molecular mechanisms that underlie recognition memory, with the aim of addressing important questions mentioned. The Research Topic comprises nine research papers and three review articles that explore a wide range of studies, from development to gene mutation, showcasing the diversity in this field.

How do animals acquire the ability to differentiate and memorize various features of objects? The research paper, by Asiminas et al. addresses this issue. The authors found that rats were able to form memory for objects as early as 4 weeks old, for contexts at 5 weeks, and for object-location-context association at 7 weeks. Importantly, a similar developmental trajectory of these memories was identified among three rat strains (Lister Hooded, Long Evans, and Sprague Dawley). These findings implicate that the PRC, lateral entorhinal cortex, and medial prefrontal cortex mature around the 4th, 5th, and 7th postnatal weeks to support object, object-context and object-location-context memories, respectively.

Does sex play a role in recognition memory? The review article, by Becegato and Silva, attempts to answer this question. While female rats are often excluded from object recognition studies due to concerns related to estrous cycle, and may be subject to biases of inadequate memory capabilities, the authors suggest that male and female rats show comparable performance in most object recognition tests.

How does information load interact with schema and episodic memories? The research paper by Harkotte et al. investigates the influence of information load on the formation of schema and episodic memories. In the elaborated version of the object-place recognition paradigm, rats were asked to learn either a low or high information load of objects and places. Rats that underwent the high information load had better schema memory for the spatial rule, while those that learned the low information load had better memory for individual episodes. The contrasting outcomes could indicate a competitive relationship between schema and episodic memory formation dependent upon the encoded information load.

How do different neurochemical systems modulate recognition memory? One review article authored by Okada et al. describes that object identity and location memories are associated with the cholinergic circuits of the nucleus basalis magnocellularis-cerebral cortices and the medial spetum/ventral diagonal band of Broca-HPC/parahippocampus, respectively, which might underlie cholinergic pathology in dementia. Meanwhile, the other review article authored by Osorio-Gómez et al. discusses the role of dopamine (DA) in recognition memory, with the suggestion that DA regulates plasticity-related mechanisms that facilitate memory consolidation and persistence, thereby enhancing perceptual salience in recognition memory, regardless of the initial sensory perception. DA also modulates memory consolidation in the rat anterior retrosplenial cortex (aRSC), as indicated by the research paper of de Landeta et al. Post-sample infusions of SCH23390, a DA D1/5 receptors antagonist, into the aRSC, or that of muscimol, a GABA_A_ receptors agonist, into the ventral tegmental area (VTA), induced object memory deficits tested 24 h later. The VTA-muscimol effects can be counteracted by aRSC infusions of SKF38393, a DA D1/5 receptors agonist. Thus, VTA might modulate object memory consolidation through the aRSC DA D1/5 receptors.

How do the cellular mechanisms of the HPC respond to the spatial properties of objects? The research paper, by Neves et al. records electrophysiological responses of the hippocampal dentate gyrus (DG), CA1, and CA3 in freely moving rats during short- or long-distance exploration between objects. Object exploration itself was linked to theta oscillations (6–12 Hz) in all the regions. Long-distance object exploration elicited higher theta power and theta-gamma phase coupling in the DG compared to exploration between neighbored objects. Stationary object exploration produced higher theta power in CA3, which correlated with CA1 gamma power. Hippocampal theta and gamma oscillations may underlie the spatial discrimination of objects into memory processing.

How do the molecular mechanisms of the HPC and PRC regulate recognition memory? The research papers, by Outram et al. and by Augereau et al. study the role of protein kinase Mζ (PKMζ) in the rat PRC concerning object memory maintenance. Post-sample (1 day, but not 6 days, later) infusions of a zeta inhibitory peptide (ZIP) that inhibits the activity of PKMζ into the PRC disrupted memory for discriminating objects, but not their places. The infusions of ZIP into the HPC produced opposite effects. Additionally, PRC ZIP infusions did not influence the perceptual ability of sensing different objects. Furthermore, blocking AMPA receptors endocytosis reversed the effects of ZIP infused into the PRC. The impairment of the long-term potential mechanism by ZIP could account for the PRC-dependent object memory maintenance. In addition, Girado et al. evaluates how the PRC endocytosis and brain derived neurotrophic factor (BDNF) affect object memory consolidation. Post-sample PRC infusions of a dynamin endocytosis function-blocking peptide disrupted object memory tested 24 h later when similar, but not dissimilar, objects were presented. Similar effects were shown when a TrkB (BDNF receptor) antagonist, ANA-12, was infused into the PRC before the learning trial. Moreover, the impairment induced by endocytosis blocking can be neutralized by BDNF infusions into the PRC. Lastly, a functional interaction effect was found between endocytosis and BDNF using a disconnection approach targeting the PRC. The PRC endocytosis interfaces with BDNF in terms of memory consolidation on similar objects.

Rossato et al. also examines the hippocampal role of c-Jun N-terminal kinases (JNK) that phosphorylates the transcription factor c-Jun in association with stress and memory in object memory consolidation and reconsolidation. Infusions of the JNK inhibitor SP600125 into CA1, 5 min, but not 6 h, after the training impaired object recognition memory. JNK inhibition did not affect the fear memory assessed by the step-down avoidance inhibitory task. The SP600125 effects were similarly shown in the reconsolidation test. Hippocampal JNK activity is important for the processes of object consolidation and reconsolidation in a time-dependent manner.

Does mutation of a disorder-relevant gene affect recognition memory? Pinizzotto et al. explore the behavioral phenotype of rats with a knockout of the phosphatase and tensin homolog-induced putative kinase 1 gene (*Pink1*) that associates with Parkinson's disease. The *Pink1* knockout rats consistently exhibited deficits in novel object, object place and object-in-place memories across ages (most cases after 5 months old). Importantly, these cognitive and memory impairments can precede the onset of confounding factors of affect and motor disturbances. The *Pink1* knockout rats could serve as a tool for investigating the cognitive neuropathology of Parkinson's disease and other neurodegenerative disorders.

This Research Topic addresses fundamental questions about the role of neurodevelopment, sex, and distinct neurochemical systems, cellular, and molecular mechanisms underlying recognition memory in the HPC and PRC. For example, memory consolidation within the PRC depends on the interaction between BDNF and endocytosis, whereas PKMζ and AMPA receptor endocytosis are associated with long-term memory maintenance. Future experiments should explore the integrated relationship between neurochemical, cellular, and molecular mechanisms across neurodevelopment, sex, and memory stages. For instance, in memory consolidation, understanding how HPC and PRC oscillations interact with dopamine, protein kinases, and endocytosis would be valuable. Additionally, single-cell sequencing of activated neuronal populations (conceptually the engram cells) during spontaneous object exploration paradigms could establish cellular and molecular mechanisms underlying recognition memory. Ultimately, this Research Topic aims to inspire new investigations and encourage collaboration to advance our knowledge of recognition memory and its significance in health and disease.

## Author contributions

OC wrote the main editorial. FB, MI, JA, and J-SL modified it. All authors contributed to research papers and review articles invitation. All authors contributed to the article and approved the submitted version.

